# Pituispheres Contain Genetic Variants Characteristic to Pituitary Adenoma Tumor Tissue

**DOI:** 10.3389/fendo.2020.00313

**Published:** 2020-05-22

**Authors:** Raitis Peculis, Ilona Mandrika, Ramona Petrovska, Rasma Dortane, Kaspars Megnis, Jurijs Nazarovs, Inga Balcere, Janis Stukens, Ilze Konrade, Valdis Pirags, Janis Klovins, Vita Rovite

**Affiliations:** ^1^Department of Human Genetics and Molecular Medicine, Latvian Biomedical Research and Study Centre, Riga, Latvia; ^2^Department of Pathology, Pauls Stradiņš Clinical University Hospital, Riga, Latvia; ^3^Department of Endocrinology, Riga East Clinical University Hospital, Riga, Latvia; ^4^Department of Internal Medicine, Riga Stradinņš University, Riga, Latvia; ^5^Department of Neurosurgery, Pauls Stradiņš Clinical University Hospital, Riga, Latvia; ^6^Clinic of Internal Medicine, Pauls Stradiņš Clinical University Hospital, Riga, Latvia; ^7^Faculty of Medicine, University of Latvia, Riga, Latvia

**Keywords:** pituispheres, pituitary adenoma, tumor sequencing, whole exome sequencing, pituitary adenoma cultures

## Abstract

The most common type of pituitary neoplasms is benign pituitary adenoma (PA). Clinically significant PAs affect around 0.1% of the population. Currently, there is no established human PA cell culture available and when PA tumor cells are cultured they form two distinct types depending on culturing conditions either free-floating aggregates also known as pituispheres or cells adhering to the surface of cell plates and displaying mesenchymal stem-like properties. The aim of this study was to trace the origin of sphere-forming and adherent pituitary cell cultures and characterize the potential use of these surgery derived cell lines as PA model. We carried out a paired-end exome sequencing of patients' tumor and germline DNA using Illumina NextSeq followed by characterization of corresponding PA cell cultures. Variation analysis revealed a low amount of somatic mutations (mean = 5.2, range 3–7) in exomes of PAs. Somatic mutations of the primary surgery material can be detected in the exomes of respective pituispheres, but not in exomes of respective mesenchymal stem-like cells. For the first time, we show that the genome of pituispheres represents genome of PA while mesenchymal stem cells derived from the PA tissue do not contain mutations characteristic to PA in their genome, therefore, most likely representing normal cells of pituitary or surrounding tissues. This finding indicates that pituispheres can be used as a human model of PA cells, but combination of cell culturing techniques and NGS needs to be employed to adjust for disability to propagate spheres in culturing conditions.

## Introduction

The pituitary gland is an important controller of the endocrine system regulating a range of physiological processes such as metabolism, reproduction, stress responses, growth, and others via secretion of specific hormones (1). Although pituitary has a volume of ¾ of a cm^3^ (2), it harbors up to 15% of all intracranial neoplasms and the vast majority of those are relatively benign (3).

Clinically significant pituitary adenoma (PA) is a rare non-metastasizing endocrine tumor affecting ~1 person out of 1,000–1,300 in general population (3, 4). Main clinical manifestation of PA is hormonal disturbance (usually, oversecretion of certain hormone) but around 30% of PA are non-functioning (NFPA), not secreting any hormones in noticeable amount and their impact on patient's health is due to the mass effect (especially affecting *chiasma optica, dura mater*, and portal arteries). In 2017, additional classification of PA was implemented based on the cell lineages which are present in the tumor (5, 6). PAs are treated via surgical resection or with drugs targeting dopamine and somatostatin receptors in prolactin-secreting and growth hormone-secreting adenomas, respectively (7, 8).

The small size of PA and their inaccessibility for biopsies coupled with lack of model cell lineage of human PA and overall PA heterogeneity among patients creates a situation where identification of novel drug targets and screening of existing compound libraries are greatly restricted. PA is a tumor of monoclonal origin (9, 10) and pituitary itself as well as PA has been reported to contain a detectable amount of stem cells and cancer stem cells (CSC) (11–14), respectively. CSC, once thought to be located only in malignant tumors, are increasingly considered important players in the development of PA. It is also proposed that there might be different types of CSC populations in PAs. Using non-adherent stem cell-permissive medium many authors have demonstrated free-floating sphere formation, that might represent PA CSC. Mostly, these cell populations expressed stem cell characteristic markers OCT4, CD133, nestin, SOX2, CXCR4. Although there are conflicting reports about their ability to self-renewal and differentiation into hormones producing cell types, as well as their ability to produce secondary tumors as xenografts (15–18), the cells isolated from pituitary and PA were capable of forming free-floating cell aggregates (pituispheres) *in-vitro* in all of these studies. As there are still no human-based model system of PA, the pituispheres, if truly representing PA CSC, might become a useful model system for PA tumorigenesis studies.

Another type of presumably CSC population has been isolated from PA tissue using adherent cell culturing method that has led to the isolation of population of mesenchymal stem-like cells (MSC). These cells fulfilled the criteria of multipotent mesenchymal stem cells described in (19), (1) were adherent in standard culture conditions, (2) expressed CD73, CD90, CD105, (3) were able to differentiate into osteoblasts, adipocytes, chondroblasts. However, these mesenchymal stem-like cells had limited self-renewal potential and were not able to differentiate in hormone-producing cells (20, 21). The question remains open what could be the role of multipotent mesenchymal stem cells in PA pathogenesis and whether these cells contribute to tumor formation or are of extra-tumoral functionality and might contribute to the tumor microenvironment.

Both pituispheres in stem cell-permissive medium and MSC in serum supplemented media are used for PA studies (22–25), additionally other approaches like three dimensional cell cultures in alginate beads or h7H humanized PA culturing methods are developed (26, 27), but how well the obtained cell cultures actually represent the tumor tissue is not clear.

In the presented study, we investigated genetic makeup of pituispheres and adherent mesenchymal cells derived from the same tumor in a group of five PA patients. Our objective was to investigate if free-floating spheres represent PA tumor-derived cells at the genomic level and if they are different from extra-tumoral pituitary cells present in PA resection material. These findings will help to evaluate the use of spheres as a human model of PA cells for in-depth functional research of PA.

## Materials and Methods

### PA Sample Collection

PA patients were recruited to the Genome Database of Latvian Population (LGDB) that is government-funded national biobank, blood samples were taken and processed according to the protocol described in (28), all biobanking activities and research in this article complies with the Declaration of Helsinki. Two informed consents were obtained from each patient after full explanation of the purpose and nature of all procedures used, broad consent for LGDB for use of biological material, and medical data for human health and hereditary research, and project-specific consent to the research of pituitary tumors. Both biobank and PA research study have been approved by the Central Medical Ethics Committee of Latvia (protocol No. 22.03.07/A7 and 2/18-02-21, respectively). Samples of human pituitary adenoma surgery material from the patients were obtained immediately after transsphenoidal resection at the Pauls Stradins Clinical University Hospital (Riga, Latvia). PA tissue samples (after resection) were carefully divided into two parts. One part was submerged in RNAlater™ Solution (Thermo Fisher Scientific, USA) for DNA/RNA extraction, and another part was immersed in Dulbecco's Modified Eagle Medium (DMEM) (Thermo Fisher Scientific, USA) containing 1x penicillin/streptomycin solution (GIBCO, USA) for cell culture development.

### Culturing of PA Tissue Material

PA tissue samples were processed within 12 h after surgery. Tissue material was mechanically cut into small pieces and washed in DMEM with 1x Antibiotic-Antimycotic solution (Thermo Fisher Scientific, USA). Enzymatic dissociation with Accutase solution (Thermo Fisher Scientific, USA) was performed for 20 min at 37°C on a rotating platform in a humidified atmosphere maintained at 5% CO_2._ At the end of incubation, the cells were pelleted by centrifugation (5 min, 360 g). To reduce red blood cell contamination, the cell pellet was incubated with red blood cell lysis buffer (154 mM NH_4_Cl, 10 mM KHCO_3_, 0.1 mM EDTA, pH 7.4) for 10 min. The sample was centrifuged, and the cell pellet was washed twice to remove red blood cell debris. The final pellet was divided into two parts. To obtain PA tissue-derived free-floating spheres cells were grown in DMEM-F12 (Thermo Fisher Scientific, USA), containing 1x penicillin/streptomycin solution, 20 ng/ml epidermal growth factor (EGF) (Sigma-Aldrich, Germany), 10 ng/ml basic fibroblast growth factor (bFGF) (Sigma-Aldrich, Germany) and 1x B27 supplement (GIBCO, USA), the primary pituispheres were cultured in sphere promoting media up to 2 months. To obtain MSC culture cells were grown in DMEM-F12 supplemented with 10% Fetal Bovine Serum (FBS) (Thermo Fisher Scientific, USA), 1% ITS (Corning, USA), and 100 μg/ml primocin (InvivoGen, USA) until reaching confluency, MSC culture were propagated and passaged at least 2–6 times. All cell culture cultivations and incubations were done at 37°C, 95% air, and 5% CO_2_.

### Immunofluorescent Staining of Cultured Cells

Spheres were harvested on microscope slides by cytospin centrifugation at 110 g for 6 min and fixed in 50/50 acetone/methanol for 20 min at −20°C. MSC cells adhered to glass coverslips were fixed with 4% paraformaldehyde in PBS for 15 min. After fixation samples were washed three times for 5 min with PBS. In the case of intracellular markers, cells were permeabilized with 0.1% Triton X-100/PBS solution for 10 min and washed three times for 5 min with PBS. Non-specific antibody binding was blocked by incubating samples for 30 min in PBS with 2% bovine serum albumin (BSA/PBS). The cells were incubated for 1 h at room temperature or at +4°C overnight with specific primary antibodies diluted in BSA/PBS at concentrations recommended by manufacturers. The following antibodies were used: mouse anti-Pit-1 (Santa Cruz Biotechnology, sc-25258), mouse anti-nestin-Alexa Fluor 488 (Santa Cruz Biotechnology, sc-33677), mouse anti-CD15-Alexa Fluor 488 (Santa Cruz Biotechnology, sc-19648), rabbit anti-GFRa2 (Thermo Fisher Scientific, PA1-33036), synonym of SF-1—rabbit anti-NR5A1 (Thermo Fisher Scientific, PA5-25030), rat anti-SOX2-Alexa Fluor 488 (Thermo Fisher Scientific, 53-9811-82), and mouse anti-CD90-FITC (Thermo Fisher Scientific, 11-0900-81). Isotype-matched antibodies were used for negative control samples. After washing with PBS, the appropriate secondary antibodies diluted in BSA/PBS were added and the cells were incubated for 1h in the dark. The following secondary antibodies were used: goat anti-mouse IgG-Alexa Fluor 488 (Santa Cruz Biotechnology, sc 3890), goat anti-rabbit IgG-Alexa Fluor 647 (Thermo Fisher Scientific, A-21244). Samples were washed, counterstained with 4,6-diamidino-2-phenylindole (DAPI) and mounted in Prolong Diamond antifade reagent (Thermo Fisher Scientific, USA). Images were acquired on confocal microscope Leica TCS SP8 (Leica Microsystems, USA) and processed using Leica Application Suite X (LAs X) software. Spheres were considered positive for the marker if at least a few cells in the sphere demonstrated positive staining, a sphere was considered negative for the characterized marker if none of the cells in the sphere showed positive immunofluorescence. To exclude the methodological error of SOX2+ antibody we performed staining of SOX2 expressing ovarian teratocarcinoma cell line PA-1 obtaining the positive signal.

### Immunohistochemistry Staining of Paraffin-Embedded PA Tissue

Immunohistochemical analysis of paraffin-embedded PA tissues was performed as an outsourcing service in the Pauls Stradins Clinical University Hospital Institute of Pathology. The following antibodies were used: mouse anti-Growth Hormone (GH) Antibody (MA5-11926), mouse anti-Prolactin (PRL) Monoclonal Antibody (MA5-11998), mouse anti-ACTH Monoclonal Antibody (MA5-13455), mouse anti-Thyroid Stimulating Hormone (TSHb) Antibody (MA5-12159), mouse anti-Luteinizing Hormone (LHb) Monoclonal Antibody (MA5-12138), mouse anti-Follicle Stimulating Hormone (FSHb) Monoclonal Antibody (MA5-12144), mouse anti-Follicle Stimulating Hormone alpha (CGA) Monoclonal Antibody (MA1-82895), rabbit anti-SSTR2 Polyclonal Antibody (PA3-109), rabbit anti-SSTR5 Polyclonal Antibody (PA3-112), rabbit anti-AIP Polyclonal Antibody (PA5-29862), mouse anti-Cytokeratin 8 (CK8) Monoclonal Antibody (MA5-14428) from Thermo Fisher Scientific, USA, and synonym of Tpit—rabbit anti-TBX19 Antibody (HPA072686) from Atlas Antibodies, Sweden. The staining was performed on an automated Daco IHC Stain system. Protein expression was evaluated using 0–3-mark system (1 = <30% positive cells; 2 = 30–70% positive cells; 3 = >70% positive cells). Additional notes were made regarding special characteristics of protein expression patterns (whether cells with positive staining are equally scattered across the sample or arranged in groups; proteins are expressed in the cytoplasm, nucleus, cell membrane, or cytoplasmic inclusions).

### DNA Extraction

DNA was extracted from whole blood using the phenol-chloroform method in standardized biobank setting (28). PA tissue samples in RNAlater™ Solution were stored for <24 h in +4°C and after that at −20°C, until enough samples are collected for convenient DNA/RNA extraction. Twenty to thirty milligrams of tissue samples from surgery material, intended for DNA extraction, were lysed using Lysing Matrix D and FastPrep-24 homogenizer (MP Biomedicals, USA). DNA was extracted from the lysate using AllPrep DNA/RNA Mini Kit (Qiagen, Germany) following the manufacturer's instructions. The concentration of extracted DNA was measured with Qubit™ dsDNA HS Assay Kit and Qubit® 2.0 Fluorometer (Thermo Fisher Scientific, USA).

### Whole-Genome Amplification (WGA)

Spheres were used directly in whole genome amplification (WGA) reaction to obtain sufficient DNA amount for library preparation. The individual spheres were manually collected with micropipette tip under the microscope in a total volume 4 μl of PBS, frozen immediately after collection, and stored at −80°C until use. The cell lysis and whole genome amplification reactions were performed using the Repli-g Single Cell kit (Qiagen, Germany) according to the manufacturer's instructions. A negative control containing PBS instead of cells was added during WGA amplification as a control for contamination.

The concentration of WGA products was quantified with Qubit 2.0 fluorometer using a dsDNA HS Assay kit. The size distribution of the WGA products was checked using the Agilent 2100 Bioanalyzer (Agilent Technologies, USA). DNA from mesenchymal cell cultures were extracted directly without WGA using AllPrep DNA/RNA Mini Kit.

### Library Preparation and Exome Sequencing

DNA library preparation and sequencing were outsourced to commercial sequencing service provider Genera Ltd. (Latvia). DNA libraries were prepared with Illumina Nextera TruSeq Exome kit (Illumina, USA) following the manufacturer's instructions and sequencing was performed with Illumina NextSeq 500/550 High Output v2 kit (150 cycles) (Illumina, USA). Paired-end sequencing with read length 75 bp was carried out on Illumina NexSeq 500 sequencer (Illumina, USA). Sequencing was performed in two batches with all corresponding sample types from PA02, PA03, and PA05 as a first batch and PA01 and PA04 as a second batch. Due to the insufficient overall coverage for PA05 this sample was resequenced including other three types of samples from this patient to achieve coverage compatible with other samples and use this case as technical replicate for estimation of sequencing consistency.

### Data Analysis

Illumina exome target manifest TruSeq Rapid Exome TargetedRegions v1.2 (Illumina, USA) was used to define exome regions. This version of exome has a total length of 45,297,543 bp and includes 214,126 regions. Illumina Basespace Enrichment App (v3.0.0) with default settings was used for data analysis. Workflow of the Enrichment app consisted of the following major steps: (1) Aligning reads against reference genome using Isaac (2, 29) Calling small germline variants (SNVs and indels) using Starling (https://github.com/sequencing/isaac_variant_caller); (3) Annotating variants with Illumina Annotation Engine, and (4) Calculating alignment and variant calling metrics using Pluggable Universal Metrics Analyzer (PUMA).

Additional filters were then applied for vcf files with following parameters: Quality filters “PASS,” variation quality >10, does not have database ID (rs or cosm code) and alternative allele has at least four supporting reads. Then sample triplets or quadruplets were compared for unique variations and identified mutations were manually reviewed using IGV v2.24 software. During manual review variants in low mapping quality regions (average MAPQ <13), regions with an extensive mismatch (>3 in 41 bp window) and at homopolymer (*N* > 4) sites were discarded. All samples from a single patient were compared at the mutation site determined by Starling software and mutation status recorded. The data that support the findings of this study are available from the corresponding author upon reasonable request.

## Results

### Study Samples

The characteristics of PA patients enrolled in this study and recruited to the LGDB by endocrinologists are presented in Table 1. Patients for this study were recruited from PA resection surgeries performed from January 2018 to June 2018 at the Pauls Stradins Clinical University Hospital. Three of the PA patients clinically were diagnosed with somatotropinoma and two had NFPA. Mean age of PA diagnosis the studied PA patients was 49.2 years (±6.5 years).

**Table 1:** Study sample characterization.

**Sample code**	**Age at PA diagnosis**	**Clinical diagnosis**	**Pituitary cell lineage**	**Tumor size/invasiveness**	**Drug therapy before surgery**	**No. of surgeries**	**DNA samples sequenced**
PA01	40	Somatotropinoma	Pit-1	Macro	DA/SSA	1	G, S, PS
PA02	46	Somatotropinoma	Pit-1	Macro	–	1	G, S, PS, MSC
PA03	53	Somatotropinoma	Pit-1	Macro/extrasellar growth	–	1	G, S, PS, MSC
PA04	50	NFPA	SF-1	Macro/extrasellar growth	–	2, relapse after PA in 2009	G, S, PS, MSC
PA05	57	NFPA	SF-1	Macro/extrasellar growth	–	1	G, S, PS, MSC

*Macro, macroadenoma > 10 mm; SSA, somatostatin analog; G, germline DNA; S, somatic DNA; PS, DNA of pituispheres; MSC, DNA of mesenchymal stem-like cells; PA, pituitary adenoma; NFPA, non-functional pituitary adenoma*.

### Characterization of Isolated Pituitary Adenoma Cells

**Figure 1:**
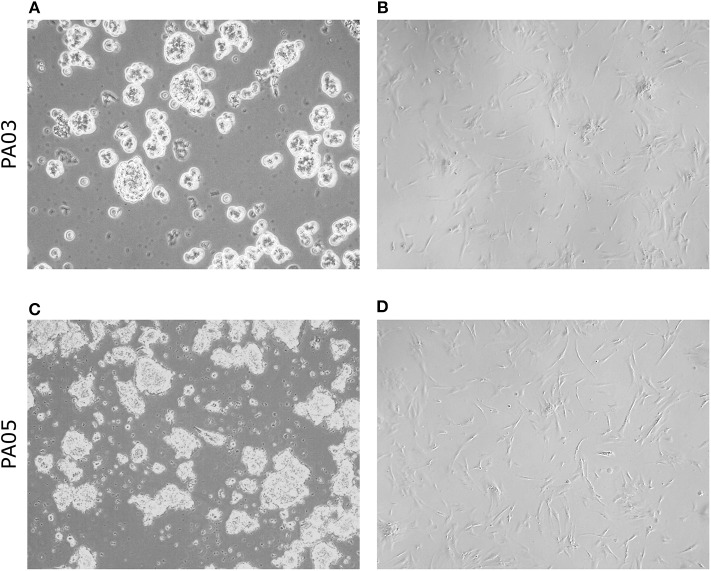
Morphology of pituispheres and MSC isolated from pituitary adenoma samples. Representative phase-contrast images from short term (4 days) cultured pituispheres **(A,C)** and first passage MSC culture **(B,D)** isolated from PA03 **(A,B)** and PA05 **(C,D)** adenoma samples. 200 × magnification of pituispheres and 400 × magnification of MSC.

Material for cell culture experiments was obtained from five resected PA tissues, representative images of PA03 and PA05 are presented in Figure 1. We managed to obtain both primary pituisphere cultures and MSC cultures from four PA tissue samples (PA02; PA03, PA04, and PA05) while for the fifth PA (PA01) sample only pituispheres were harvested due to a limited amount of resected tissue. To avoid potential fibroblast overgrowth of cell cultures at the beginning of the study the cells were grown into two parallel aliquots: one in DMEM media with D-valine, which has been reported to prevent fibroblast growth due to the absence of D-amino acid oxidase in these cells, and other in DMEM with L-valine. No substantial differences were observed between cells growing in both culture media (data not shown). The study was continued and pituispheres were cultured in sphere promoting media, collected for WGA, and exome sequencing and analyzed together with a corresponding formalin-fixed paraffin-embedded (FFPE) adenoma tissue samples for stem/progenitor and pituitary lineage-specific commitment cell markers such as SOX2, CD15, GFRα2, nestin, Pit-1, SF-1, and Tpit (Table 2).

**Table 2:** Characterization of PA tissue samples and pituispheres for hormone and cell marker expression by immunohistochemistry (IHC) and immunofluorescent analysis (IF).

**Analysis**	**Marker**	**PA01**	**PA02**	**PA03**	**PA04**	**PA05**
IHC of paraffin-embedded PA tissue	GH		3	2	0	0
	PRL		3	1	1	0
	ACTH		1	2	0	1
	TSHb		0	0	1	0
	LHb		0	1	1	0
	FSHb		0	1	0	0
	CGA		1	3	3	2
	SSTR2		0	2	2	3
	SSTR5		1	2	1	3
	AIP		2	3	2	3
	CK8 densely granulated		2	2	1	1
	CK8 sparsely granulated		2	3	0	0
	Pit-1	3	3	2	1	1
	SF-1	0	2	1	3	2
	Tpit	0	1	1	0	1
	CD15	1	1			1
IF characterization of pituishperes	GFRa2	+	+	–		+
	Pit-1	+	+	+		–
	CD15	+	+	+		+
	SF-1	+	+	–		+
	NES	–	–	–		–
	SOX2	–	–	–		–

*IHC protein expression evaluation was performed using 0–3-mark system with 1 = <30% positive cells; 2 = 30–70% positive cells; 3 = >70% positive cells, IF protein expression positive (+) if at least a few cells in the sphere were positive, negative (–) if not a single cell in a sphere expressed the marker, blank boxes: not done*.

**Figure 2:**
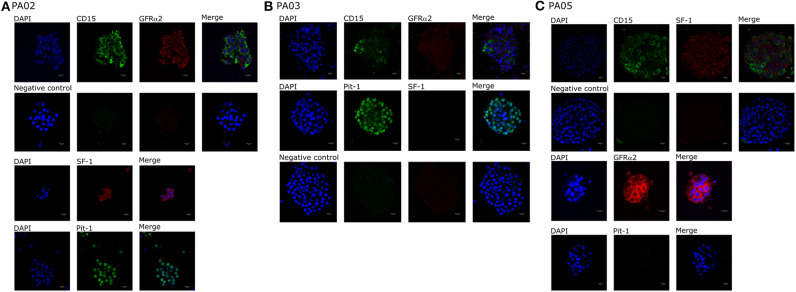
Expression of cell markers in pituispheres. Representative immunofluorescence images of **(A)** PA02, **(B)** PA03, and **(C)** PA05 pituispheres. Cells were double-stained for **(A,B)** CD15 (green) and GFRα2 (red), **(C)** Pit-1 (green), and SF-1 (red) **(B)** CD15 (green) and SF-1 (red) and PA02 pituispheres were single stained for **(A)** Pit-1 (green) and SF-1 (red) and PA05 pituispheres were single stained for **(C)** Pit-1 (green) and GFRα2 (red). Isotype-matched antibodies were used for negative controls. Cell nuclei were counterstained with DAPI (blue). Scale bar, 13 μm.

We observed CD15+ cells in PA01, PA02, PA03, and PA05 pituispheres (Figure 2) and their corresponding tissue samples mostly as isolated cells or in small clusters (data not shown).

**Figure 3:**
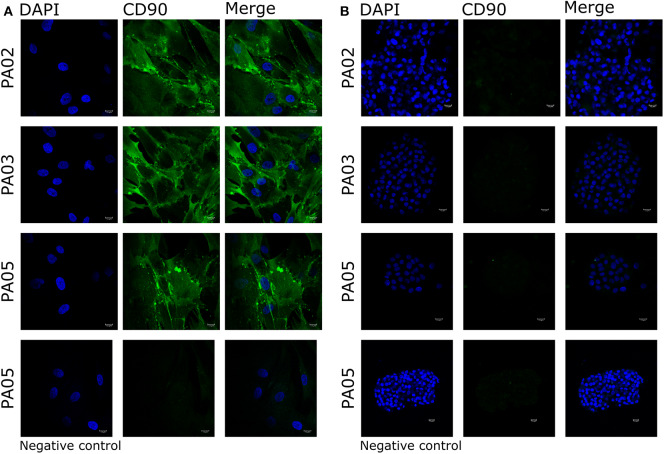
Expression of CD90 marker in pituispheres and MSC. Immunofluorescence images of PA02, PA03, and PA05 **(A)** MSC and **(B)** pituispheres stained for CD90 (green). Isotype controls were used as negative control. Cell nuclei were counterstained with DAPI (blue). Scale bar, 13 μm.

Pit-1 transcription factor was expressed in the majority of cells in all tested samples, except PA05. SF-1+ and GFRα2+ cells were detected in all tested samples except PA03. Pit-1 and SF-1 markers demonstrated comparable expression in pituispheres and FFPE of tumor tissue obtained from the same patient. None of the samples showed positive staining for nestin and SOX2 markers (Supplementary Figure 1). PA04 pituispheres were not stained due to technical problems with microscopy, PA01 was stained but only visual evaluation of the presence of the above-mentioned markers was performed (Table 2). We also evaluated expression of MSC cell surface marker CD90 in both pituispheres and MSC cultures, none of the pituispheres expressed CD90, while it was highly presented in MSC cultures (Figure 3).

### Exome Sequencing and Estimation of the Somatic PA Mutations

Libraries were successfully generated from the DNA obtained from white blood cells, fresh tumor tissue, pituispheres, and MSC for all five patients with the exception of MSC sample from PA01. Exome sequencing from white blood cells was performed to compare mutations found in tumor tissue with germline DNA and to exclude from further analysis any mutations that were found in germline DNA. Initial exome sequencing of all 19 DNA samples was performed in two sequencing runs. Individual exome sequencing statistics are available upon request.

**Figure 4:**
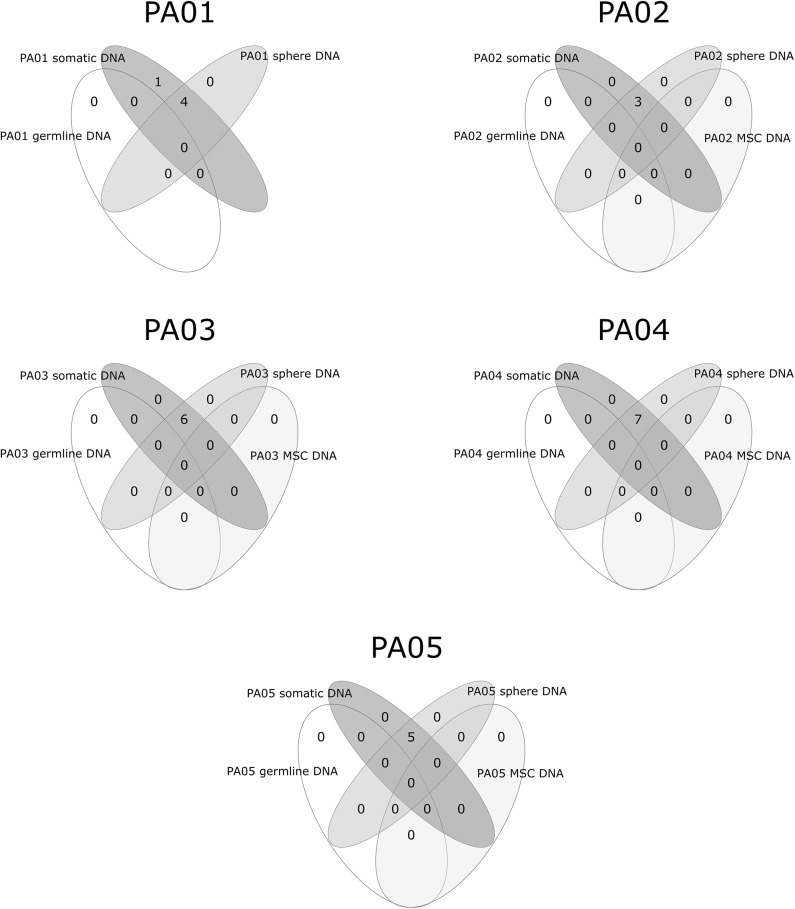
Overlapping somatic mutations in somatic tumor tissue, pituispheres, MSC, and patients' germline DNA.

Exome sequencing data analysis revealed a low amount of somatic mutations per tumor exome (mean = 5.2, range 3–7). A combined total number of somatic mutations in all five PA samples was 26 and 24 of those were unique. There was recurrent somatic DNA mutation in *GNAS* (rs11554273) more commonly known as *GNAS* 201 R > C, which was present in three PA patients (PA01, PA02, PA03). All mutations discovered in somatic DNA were also present in the DNA from pituispheres, but not in the DNA from MSC (Figure 4). A single exception was the mutation in *PLEKHH1* in PA01 that was not detected in pituispheres due to the low coverage of the corresponding region (Supplementary Table 1, Figure 4).

SNVs represented 88% (23 out of 26) of PA somatic mutations while indels were12% (*n* = 3). Four of 24 unique somatic mutations across all five samples were located on chromosome one and three somatic mutations on both chromosome three and six. Somatic mutation rate varied from 0.07 (PA02) to 0.15 (PA03) mutations per/Mb of the exome. Transition to transversion ratio of single nucleotide somatic mutations is 2.14:1. The overall fractions of mutated reads from a total number of reads for the corresponding site were 37 and 32% for materials from tumor tissue samples and pituispheres, respectively. There was a slight variation among patients for this ratio ranging from 32 to 42% for tumor tissue sample and 29 to 50% for pituispheres (Supplementary Table 1).

The most common consequence of the discovered SNVs was a change of amino acids in the protein-coding canonical transcript of the gene (*n* = 15), five variants were synonymous and three were located in introns. Two indels are causing stop gain and occur in 3rd and 24th exons in *HDGF* and *ZCCHC11*, respectively. The third indel was inframe in *LOR* and resulted in a gain of five amino acids in the second of two exons, altering protein sequence. Eight of 24 unique SNVs were predicted deleterious by SIFT and probably damaging by PolyPhen prediction algorithms and seven of those has CADD Phred score above 20 (range 20.8–35.0). The summary of detected mutation characteristics in canonical transcripts can be found in Table 3. Detailed information on consequences of detected somatic mutations across all Ensembl transcripts is provided in Supplementary Table 2. Several somatic mutations had dbSNP rs code assigned to them. Supplementary Table 3 contains information about these SNV in human germline DNA.

**Table 3:** Characteristics of detected mutations in somatic tumor DNA and pituispheres.

**Patient**	**CHR**	**Start position**	**Reference/** **alternative** **allele**	**dbSNP no**.	**Consequence**	**Gene symbol**	**Localization in exon or *intron*|total no. of gene exons or *introns***	**Protein position|amino acid change**	**Strand**	**CADD PHRED**
PA01	1	156714911	GA/G	–	Frameshift	HDGF	3|6	80|F/X	−1	–
	3	113012861	ACTTAG/A	rs549443662	Intron	WDR52	*34|34*	–	−1	–
	6	29141632	T/C	–	Missense	OR2J2	1|1	74|Y/H	1	23.7
	14	68028745	G/A	rs780838899	Synonymous	PLEKHH1	6|29	166|A	1	9.941
	20	57484420	C/T	rs11554273	Missense	GNAS^†^	8|13	844|R/C	1	35
PA02	5	36064400	G/A	rs372124401	Synonymous	UGT3A2	2|7	49|H	−1	2.654
	13	76407224	C/G	–	Missense	LMO7	13|27	763|T/R	1	17.5
	20	57484420	C/T	rs11554273	Missense	GNAS^†^	8|13	844|R/C	1	35
PA03	1	160580606	G/A	–	Intron	SLAMF1	*6|6*	–	−1	1.52
	2	90229251	T/A	rs748067626	Missense	IGKV1D-42	2|2	24|I/N	1	23.2
	3	169802162	T/C	–	Synonymous	GPR160	4|4	134|C	1	0.067
	4	85693997	G/T	–	Missense	WDFY3	30|68	1614|L/I	−1	18.33
	6	111499541	G/T	–	Intron	SLC16A10	*3|5*	–	1	2.55
	20	57484420	C/T	rs11554273	Missense	GNAS^†^	8|13	844|R/C	1	35
PA04	1	52911473	T/TGG	–	Frameshift	ZCCHC11	24|30	1269–1270|–/X	−1	–
	2	65541087	C/A	rs146072471	Missense	SPRED2	6|6	269|V/L	−1	20.8
	6	56422247	T/C	–	Missense	DST	40|84	2214|N/S	−1	6.265
	16	11541848	G/A	–	Synonymous	CTD-3088G3.8	30|50	1475|L	−1	8.094
	17	18874965	C/T	rs138715863	Missense	FAM83G	6|6	727|V/I	−1	0.818
	19	6906514	C/T	rs371881714	Synonymous	EMR1	9|21	340|T	1	9.185
	19	23544625	C/T	rs403356	Missense	ZNF91	4|4	386|A/T	−1	9.578
PA05	1	153234011	A/ACTCTG GCGGCGG	rs11272549	Protein altering	LOR	2|2	196|C/SLAAG	1	–
	3	167183317	C/A	rs375412954	Missense	SERPINI2	5|10	208|G/V	−1	25.6
	14	91211257	A/G	–	Missense	TTC7B	4|20	152|L/S	−1	25.6
	19	38997556	C/T	–	Missense	RYR1^‡^	57|105	2927|L/P	1	28.6
	X	86880668	T/A	–	Missense	KLHL4^‡^	6|11	399|L/H	1	28.1

CHR, chromosome;

† somatic variant previously detected in PA,

‡ *in these genes other somatic variants have been previously detected in independent PA studies*.

## Discussion

So far two distinct cell populations have been isolated from human PA with an attempt to develop cell cultures for PA *in-vitro* model. First, spheres, in some cases carrying CSC characteristics and, second, MSC having self-renewal potential but not being able to differentiate in hormone-producing cells. No conclusive answer has been obtained so far on the contribution of both of these cell types in PA development. In this study we have, for the first time, performed exome analysis to trace genetic characteristics of pituispheres and MSC, and have shown that MSC does not carry tumor-specific mutations but resemble genetic makeup of somatic body cells, therefore representing cell lineage different from cells with the PA origin.

Pituispheres have been shown to be the most likely candidate to harbor CSC that could initiate PA development. All studies performed have demonstrated that dissociated single cell can regrow in new spheroid indicating stem-like self-renewal capacity (15–17). Pituisphere derived cells are also able to differentiate in hormone-producing cells in all described studies (15, 17, 18, 30), but conflicting results have been demonstrated to the potential of these cells to form tumors. In most of the studies, the sphere cell-derived xenografts were able to initiate tumor formation in mice (15, 16, 18), but in some cases, this was not observed (17). Interestingly, in the zebrafish model transplanted cells manifested angiogenic properties, that complement discussion that in fact, CD133+ cells could be more related to tumor neovascularization (31).

Our data show that pituispheres carry the mutations found in PA resection material, therefore, the cells with sphere-forming potential likely constitute the mass of the tumor. Two models of PA pathogenesis have been proposed in literature (1) traditional CSC model when the mutation sustaining CSC proliferate and differentiate forming the tumor and (2) paracrine tumorigenesis model when CSC carrying mutations by it's paracrine signaling affect other cells in transforming them to CSC (13, 32). Our results do not exclude any of these models, but points toward the paracrine model due to the sphere immunofluorescence analysis discussed further and taking into account also the fact in literature reports that initiation of tumor formation in xenografts is limited but further investigations are needed to confirm this hypothesis.

It has been proposed that MSC, another cell lineage obtained from PA surgery materials might represent CSC in PAs (13, 20, 33). These cultures have some self-renewal capacity but are not able to differentiate into hormone-producing cells (21) and some investigations have also observed that SSA have an antiproliferative effect on MSC (33). Our results clearly show that MSC though present in PA do not carry any genetic mutations present in the majority of tumor cells and are of different origin. MSC is also unlikely to be related to PA initiation, although the supportive role in tumor microenvironment development is not excluded. Thus, if SSA causes an antiproliferative effect on MSC, this is more likely to be because of intrinsic cell properties and not because of relation to pituisphere forming cells.

Regarding cell lineage determinants of PA classification, recent findings have demonstrated that somatotroph adenomas with GNAS wild-type genetic background can express SF1 and that actually driver alterations like USP8 and GNAS in PA can induce changes in tumor transcriptome profiles that could cause clinical characteristics that deviate from WHO 2017 cell lineage classification system (34). In this study, immunohistochemistry analysis of the paraffin-embedded PA tissues demonstrated that predominant PA cell lineage markers (Pit-1, SF-1, Tpit) in the tissue corresponded to the PAs clinical characteristics regarding hormonal type. In all tested pituispheres, we detected CD15 that has been proven to be expressed in PA initiating cells (30), strongly suggesting their contribution to tumor formation. We also observed GFRa2 expression in three of the four analyzed samples. GFRa2 have been proposed to be one of the markers for stem cell fraction in pituitary and also shown to be expressed in pituispheres (35) that is supported by our findings as well. This indicates that the obtained pituispheres have specific characteristics of pituitary adenoma and it is not likely that cells in spheres could come from non-tumorous tissue counterparts.

We did not, however, observed nestin and SOX2 expression in any of the analyzed pituispheres. Although it is generally believed that these stemness markers should be present in PA CSCs supported by many reports (13, 32), there is evidence that points toward to more complex role of these factors in PA development. For example, nestin has been overall demonstrated to co-express with CD133, that is believed to be a potential PA CSC marker (13, 32). More recent studies implicate CD133+ cells to function as endothelial progenitor cell and have demonstrated the role of this factor in PA neovascularization (18, 31) possibly being crucial for supporting tumor formation by ensuring blood supply, but not initiating tumor formation *per se*. If true, this could explain why in our study we did not observe nestin in pituispheres. The information in the literature about cell fractions that express nestin but do not express CD133 in PA is scarce as potentially either co-expression has been demonstrated or only nestin analysis has been performed. Therefore, it is hard to evaluate the supporting role of nestin-expressing cell fraction and further analysis is needed.

SOX2 have been demonstrated to be expressed in pituispheres in several other studies (18, 30), together with the observation that SOX2 expression is more pronounced during the stemness state and decreases when cells start to differentiate (18). It has also been shown that SOX2+ cells can differentiate in all hormone-producing cell types of pituitary in mice model and that SOX2+ cells with altered β-catenin expression contribute to tumor formation in a paracrine manner but do not constitute cells of the PA tissue mass (36). This has been also supported by the fact that no specific Wnt signaling upregulation was observed in PA by qPCR and immunohistochemistry (37). As the pituispheres obtained by us did not express SOX2, but were composed of the cells representing the overall tumor mass as indicated by our exome sequencing data, the results of this study reinforces above mentioned hypothesis (36, 37) that SOX2+ cells in pituitary can contribute to PA development but do not form tumor tissue themselves.

Regarding the possible role of identified mutations, seven genes across all PA patients have a somatic mutation (SNV) with CADD Phred score above 20 (range 20.8–35.0) meaning that a variant is among the top 1% of deleterious variants in the human genome (38). PA01 has two somatic SNV with CADD Phred score above 20, located in *OR2J2* (23.7) and *GNAS* (35.0). PA04 and PA02 have one somatic SNV each, localized in *SPRED2* (20.8) and *GNAS* (35.0), respectively. PA03 again has two: *IGKV1D-42* (23.2) and *GNAS* (35.0) and PA05 have three highly ranked deleterious variants located in *SERPINI2* (25.6), *TTC7B* (24.6), and *KLHL4* (28.1). Interestingly this reminds of Knudson's two-hit hypothesis (39), that corresponds finding at least two deleterious variants in most tumors in our study. For the genes other than *GNAS* and *SERPINI2*, possible contribution to PA is unclear (40). Somatic variants were also checked in COSMIC and ExAc databases, but none has previously demonstrated any significant functional effects in relation to PAs or other tumors. To further understand effects of these alterations functional studies should be performed.

Special interest deserves *KLHL4* (Kelch-like 4) gene which has been reported previously to have a somatic mutation in PA. Newey et al. who also investigated exomes of non-functioning PAs, detected p.KLHL4 V481M (41), while mutation from PA05 is located in 6th instead of 7th exon and changes L399H. *KLHL4* encodes a protein of unclear function and GO term suggests actin-binding as a molecular function implying participation in cytoskeleton building or regulation (42, 43). We also discovered mutation in *RYR1* in PA05 sample, other mutations in this gene have been previously found in two independent PA tumors (44, 45). The function of *RYR1* is related to inner cell Ca^2+^ flow regulation, the dysfunctions of this factor could lead to heart conditions, myopathies, and neurodegenerative disease (46), how this gene could affect PA development needs to be further investigated. It should be noted that shared mutated genes across PA patients are quite rare in the current literature, most somatic variants in PAs are characteristic to the individual tumor and only some overlapping mutations have been reported in *GNAS, USP8*, and in rare cases the same mutated genes are found in two individual tumors (44, 45, 47).

Non-sense mutations were found in *HDGF* and *ZCCHC11* genes in PA01 and PA04, respectively. *HDGF* (*Heparin Binding Growth Factor*) encodes a member of the hepatoma-derived growth factor family that may be involved in proliferation and differentiation of the cell. It has been observed that overexpression of this gene stimulates the growth of different tumors. *ZCCHC11* or *TUT4* is involved in mRNA degradation and miRNA suppression. Currently, it is unclear if and how a loss of function of any of these two genes could contribute to PA development or growth.

Exome data of three tumors also show the presence of GNAS mutations that are identified in 40% of growth hormone-secreting PAs and in lower frequency also in other PA types (48–50). It has also been shown that GNAS mutations have an impact on clinical features of acromegaly (51), to further study how these mutations affect functionality of pituispheres extended investigation should be performed.

A limitation of this study is the use of NGS for genome sequencing only, thus restricting our ability to explore the functional properties of the analyzed material. Analysis of RNA expression especially applying single-cell RNA-seq technique would provide a better understanding of pituispheres composition and function of different cell types. Another limitation is the small number of samples used in the study, however, our main goal was to show the presence of the somatic mutations in spheres and their absence in the mesenchymal cells this has been convincingly demonstrated by the NGS. Although the results demonstrate proof of principle, it is also clear that increased number of similar sample cases would strengthen our observation.

Both, pituispheres and MSC are being used in research to investigate functionality and drug response of PA tumors (22–25, 33), including the development of new PA cell models (26, 27). Therefore, it is highly important to evaluate whether the cellular content of the cell culture models represents original PA tumor that can be achieved by NGS using a similar approach as we have shown in our study.

In conclusion, we demonstrate that pituispheres containing cells correspond genetically to the PA tissues and that PA derived MSC, although easier to obtain and cultivate, represent cell lines unrelated to tumor cells. This study proposes the use of NGS analysis for research tracing the origin of pituispheres and their functionality that could help to elucidate the mechanisms and microenvironment of PA development.

## Data Availability Statement

The datasets presented in this study can be found in online repositories. The names of the repository/repositories and accession number(s) can be found below: NCBI BioProject PRJNA623684.

## Author Contributions

RPec: participated in study design, data analysis, and manuscript writing. IM: participated in study design, performed cellular experiments, participated in manuscript writing. RPet: performed cellular experiments. RD: performed cellular experiments. KM: performed cellular experiments. JN: performed immunohistochemistry staining analysis. IB: performed patient enrolment in the study. JS: performed tumor resection and tumor material obtainment. IK: participated in study design and manuscript editing. VP: participated in study design and manuscript editing. JK: participated in study design and manuscript editing. VR: participated in study design and manuscript writing.

## Conflict of Interest

The authors declare that the research was conducted in the absence of any commercial or financial relationships that could be construed as a potential conflict of interest.

## Supplementary Material

Supplementary Figure 1 Expression of nestin and SOX2 cell markers in pituispheres.

Supplementary Table 1 Read counts and percentage of alternative alleles in tumor tissue and spheres.

Supplementary Table 2 Information of mutations with dbSNP rs# code.

Supplementary Table 3 Detailed somatic mutation data across all transcripts VEP.
