# Supplementary material for: Pituispheres Contain Genetic Variants Characteristic to Pituitary Adenoma Tumor Tissue
Source: Front Endocrinol (Lausanne). 2020 May 22;11:313. doi: 10.3389/fendo.2020.00313 (PMC7256168; doi:10.3389/fendo.2020.00313)
Supplement: Supplementary Figure 1 — Expression of nestin and SOX2 cell markers in pituispheres. [file Data_Sheet_1.docx]

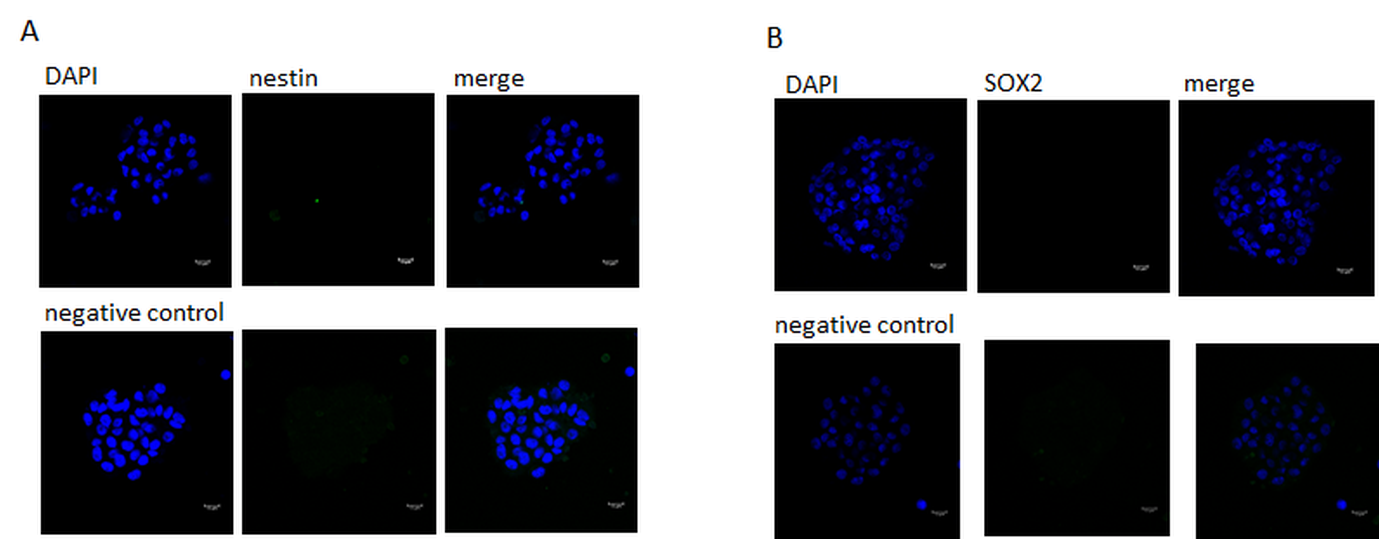


**Supplementary Figure 1.** Expression of nestin and SOX2 cellmarkers in pituispheres. Representative immunofluorescence images of PA05 pituispheres stained (A) for nestin (green) and (B) for SOX2 (green). Isotype controls were used as negative control. Cell nuclei were counterstained with DAPI (blue). Scale bar, 13 µm.
