# Supplementary material for: Pituispheres Contain Genetic Variants Characteristic to Pituitary Adenoma Tumor Tissue
Source: Front Endocrinol (Lausanne). 2020 May 22;11:313. doi: 10.3389/fendo.2020.00313 (PMC7256168; doi:10.3389/fendo.2020.00313)
Supplement: Supplementary Table 1 — Read counts and percentage of alternative alleles in tumor tissue and spheres. [file Table_1.docx]

**Supplementary Table 1. Read counts and percentage of alternative alleles in tumour tissue and spheres.**

| **Patient** | **CHR** | **Start position** | **Reference/alternative**  **allele** | **Mutation vs normal %  in tissues** | | **Mutation vs normal**  **% in spheres** | |
| --- | --- | --- | --- | --- | --- | --- | --- |
| PA01 | 1 | 156714911 | GA/ G | 14/29 48% | | 2/3 67% | |
|  | 3 | 113012861 | ACTTAG/ A | 9/27 33% | | 1/2 50% | |
|  | 6 | 29141632 | T/ C | 12/30 40% | | 1/2 50% | |
|  | 14 | 68028745 | G/ A | 17/35 49% | | 0/3 0% | |
|  | 20 | 57484420 | C/ T | 42/103 41% | | 7/12 58% | |
| PA01 total |  |  |  | **94/224 42%** | | **11/22 50%** | |
| PA02 | 5 | 36064400 | G/ A | 25/48 52% | | 4/4 100% | |
|  | 13 | 76407224 | C/ G | 46/101 46% | | 13/135 10% | |
|  | 20 | 57484420 | C/ T | 39/114 34% | | 57/78 73% | |
| PA02 total |  |  |  | **110/263 42%** | | **74/217 34%** | |
| PA03 | 1 | 160580606 | G/ A | 17/54 31% | | 7/42 17% | |
|  | 2 | 90229251 | T/ A | 20/66 30% | | 8/18 44% | |
|  | 3 | 169802162 | T/ C | 13/36 36% | | 7/18 39% | |
|  | 4 | 85693997 | G/ T | 9/36 25% | | 23/54 43% | |
|  | 6 | 111499541 | G/ T | 8/41 20% | | 8/39 21% | |
|  | 20 | 57484420 | C/ T | 57/140 41% | | 17/73 23% | |
| PA03 total |  |  |  | **124/373 33%** | | **70/224 29%** | |
| PA04 | 1 | 52911473 | T/ TGG | 10/27 37% | | 16/45 36% | |
|  | 2 | 65541087 | C/ A | 3/10 30% | | 3/7 43% | |
|  | 6 | 56422247 | T/ C | 7/29 24% | | 7/26 27% | |
|  | 16 | 11541848 | G/ A | 8/15 53% | | 7/12 58% | |
|  | 17 | 18874965 | C/ T | 9/14 64% | | 1/1 100% | |
|  | 19 | 6906514 | C/ T | 5/15 33% | | 3/10 30% | |
|  | 19 | 23544625 | C/ T | 13/39 33% | | 2/13 15% | |
| PA04 total |  |  |  | **55/103 37%** | | **39/114 34%** | |
| PA05 | 1 | 153234011 | A/G | I 2/8 25% | II 6/22 27% | I 1/19 5% | II 2/13 15% |
|  | 3 | 167183317 | C/ A | I 6/19 32% | II 41/101 41% | I 15/41 37% | II 52/116 45% |
|  | 14 | 91211257 | A/ G | I 10/22 45% | II 13/36 36% | I 2/9 22% | II 4/28 14% |
|  | 19 | 38997556 | T/C | I 9/35 26% | II 34/96 35% | I 7/13 54% | II 2/16 13% |
|  | X | 86880668 | T/ A | I 3/10 30% | II 2/27 7% | I 1/4 25% | II 1/11 9% |
| PA05 total |  |  |  | **126/376 34%** | | **87/270 32%** | |
| **TOTAL** |  |  |  | **509/1385 37%** | | **281/867 32%** | |

I – data from first sequencing run of PA05, II – data from second sequencing run of PA05.
